# Negative correlation between metabolic score for insulin resistance index and testosterone in male adults

**DOI:** 10.1186/s13098-024-01353-5

**Published:** 2024-05-23

**Authors:** ChunMei Li, Jing Xu

**Affiliations:** 1 Department of Gastroenterology, The Second Affiliated Hospital and Yuying Children’s Hospital of Wenzhou Medical University, Lucheng District, Wenzhou, Zhejiang Province China; 2 Department of Endocrinology, The Second Affiliated Hospital and Yuying Children’s Hospital of Wenzhou Medical University, Lucheng District, Wenzhou, Zhejiang Province China

**Keywords:** Metabolic score for insulin resistance; Testosterone; Insulin resistance

## Abstract

**Background:**

Insulin resistance (IR) is strongly correlated with the decreased deficiency of testosterone levels in males. The metabolic score for insulin resistance (METS-IR) index is regarded as an innovative measure for the assessment on IR. The research aims to explore the correlation between the METS-IR index and the testosterone levels in males.

**Methods:**

In this study, a cross-sectional design was made through the data obtained from the National Health and Nutrition Examination Survey (NHANES) from 2013 to 2020. Besides, the METS-IR index was derived from serum triglyceride levels, fasting plasma glucose, HDL-C and BMI.

**Results:**

A total of 2082 participants were included in the final analysis. After controlling for confounding variables, it was found that METS-IR was independently and negatively correlated with testosterone levels (β = −3.88, 95% CI = −4.49, −3.27, P < 0.001). As shown by the generalized smooth curve fitting, METS-IR had a linear correlation with testosterone levels without threshold or saturation effects, which was consistently observed across all subgroups through stratified analysis (all P > 0.05). As revealed by the analysis on the ROC curve, the area under the curve (AUC) for the METS-IR index (0.732, 95% CI = 0.705, 0.760) was significantly larger than that of homeostatic model assessment of insulin resistance (HOMA-IR), TG/HDL ratio, triglyceride-glucose index (TyG) and body mass index (BMI).

**Conclusion:**

The findings suggest a negative relationship between the METS-IR index and the testosterone levels in male adults. Furthermore, the METS-IR index demonstrates superior predictive ability for testosterone deficiency in comparison to HOMA-IR, TG/HDL ratio, TyG and BMI.

## Introduction

Testosterone, the primary male sex hormone produced by Leydig cells, plays a crucial role in various physiological processes in males, such as sexual function, metabolism, cardiovascular health, bone density, and cerebral function [1–3]. However, insufficient levels of serum testosterone in males can trigger dysfunctions in multiple organs, which are manifested as decreased libido, erectile dysfunction, and potentially exacerbating metabolic disorders such as depression and osteoporosis. which is commonly referred to as the testosterone deficiency syndrome [4–7], or male hypogonadism [8]. Testosterone deficiency is a prevalent disorder influencing approximately 7% of males aged 50 and above, and its occurrence tends to increase as the growth of age. As suggested by projections, this condition will escalate alongside the average lifespan in the forthcoming decades [9]. Furthermore, hypogonadism is common in males with type 2 diabetes mellitus (T2DM), and about one-third of males with T2DM have low serum testosterone levels [10]. Testosterone deficiency was increasingly alarming worldwide.

As a significant contributory factor, IR plays a crucial role in the development and advancement of cardiometabolic diseases. Furthermore, it has been observed that hypogonadism is prevalent among individuals with metabolic comorbidities, including DM and obesity [11]. In spite of this, lots of studies have underscored the close correlation between IR and testosterone deficiency. According to the study of Souteiro et al., IR can serve as the primary risk factor for low testosterone levels in obese males, and some males suffering from testosterone deficiency exhibit a higher IR index than those with mild DM [12]. In addition, a deficiency or decrease in testosterone can be linked to the development of metabolic disorders, including increased IR and visceral lipids [13]. Moreover, the bidirectional relationship between metabolic disorders and hypogonadism has been established in cases of functional hypogonadism and late-onset hypogonadism [14]. Therefore, it is a prominent research field to investigate the correlation between IR and male testosterone.

The complexity, time-consuming nature, and limited applicability of traditional IR evaluations, such as the HOMA-IR and hyperinsulinemic-euglycemic clamp (HIEC), have prompted the need for alternative approaches. For example, Bello-Chavolla et al. introduced a pioneering non-insulin-dependent metabolic score of insulin resistance, known as METS-IR, integrating fasting plasma glucose, triglycerides, HDL-C, and BMI as indicators of nutritional status [15]. Meanwhile, this novel scoring system has demonstrated superiority over other non-insulin-based markers of IR (e.g. TyG and TG/HDL ratio) [15]. Although the relationship between METS-IR and various diseases, such as DM [15], non-alcoholic fatty liver disease (NAFLD) [16], hyperuricemia[17], hypertension [18] and metabolic syndrome [19], has been documented in previous research, with a lack of research investigating the potential links between METS-IR and testosterone at present.

This study aims to examine the correlation between the METS-IR index and testosterone levels in males based on a nationally representative sample of American male adults, and assess the predictive capacity of the METS-IR index in identifying testosterone deficiency.

## Methods

### Study population

In this study, the data was collected from NHANES (2013–2020), a comprehensive survey conducted by the National Center for Health Statistics (NCHS). NHANES has employed a complex, stratified, and multi-stage probability sampling design to ensure the representation of the non-institutionalized population in America. Further information regarding the methodology of NHANES can be accessed at www.cdc.gov/nchs/NHANEs/.

**Fig. 1:**
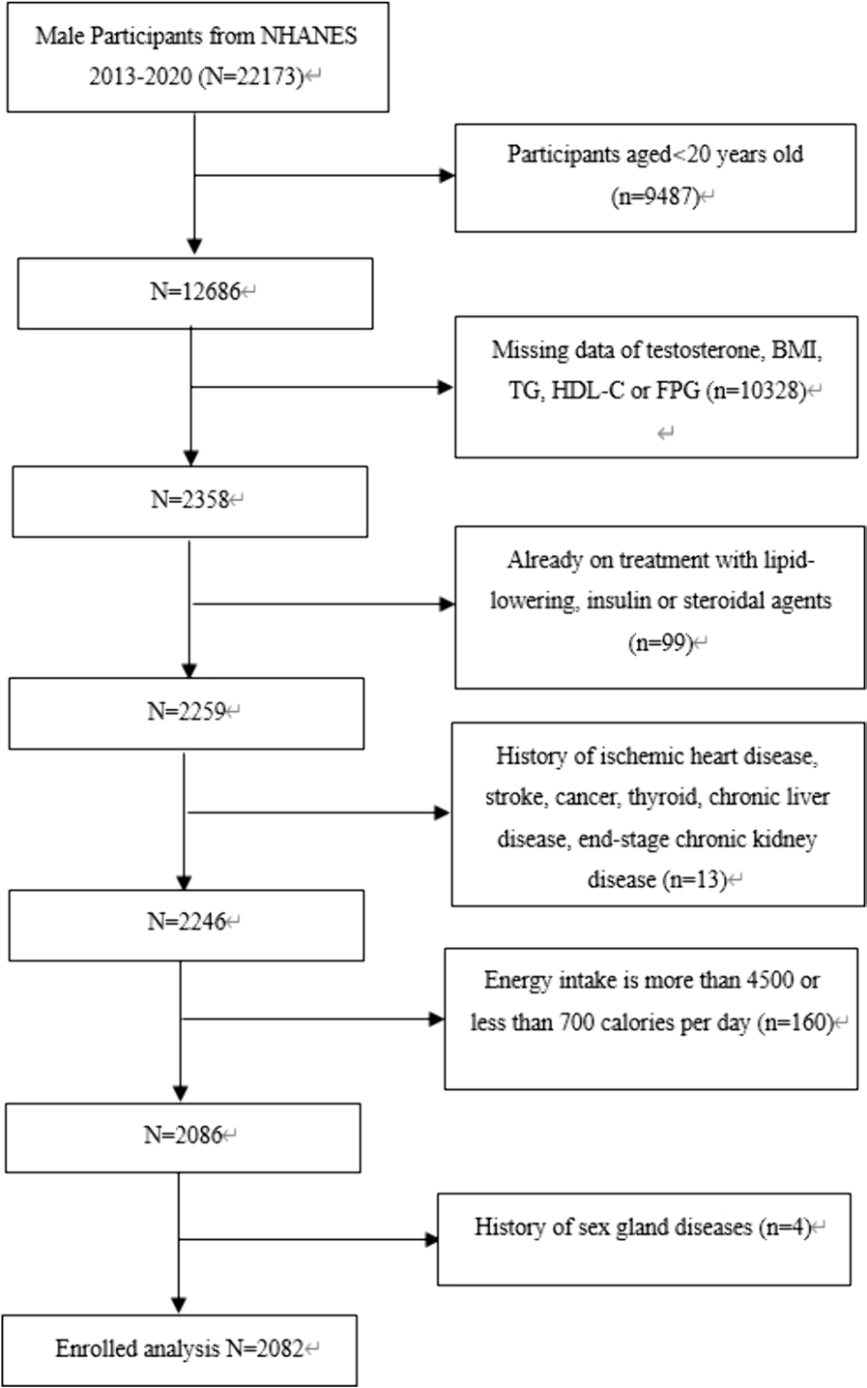
Flowchart of the sample selection from the 2013–2020 NHANES

This study focuses exclusively on males aged 20 and above (n = 12,686). Exclusion criteria were as follows for: (1) patients missing data on testosterone, BMI, TG, HDL-C or FPG, (2) patients who had already taken treatments with lipid-lowering, insulin or steroidal agents, (3) patients with a history of ischemic heart disease, stroke, cancer, thyroid, chronic liver disease, end‑stage chronic kidney disease, (4) patients whose energy intake was more than 4500 or less than 700 cal per day, (5) patients with the history of sex gland diseases. Consequently, 2082 participants aged from 20 to 80 were involved in the final analysis (Fig. 1).

The implementation of NHANES was approved by the NCHS Ethics Review Board, and all participants provided the written informed consent [20].

### Biochemical and anthropometric measurements

The following data were collected at admission, including history of DM, daily total energy intake, race, physical activity, education level and physical measurements including waist circumference (WC), height, systolic blood pressure (SBP), diastolic blood pressure (DBP) and weight, etc. Obesity was defined as BMI ≥ 30 kg/m^2^, non-obesity was defined as BMI < 30 kg/m^2^.

Total cholesterol (TC), glycosylated hemoglobin (HbA1c), LDL-C, fasting insulin (FINS), uric acid (UA), fasting plasma glucose (FPG), TG, alanine aminotransferase (ALT), aspartate aminotransaminase (AST), estradiol (E2), creatinine, sex hormone binding globulin (SHBG), albumin, testosterone and HDL-C in blood samples were collected. Moreover, the total number of missing values was less than 3%. Multiple imputations had been performed for missing values. The detailed measuring method and acquisition of each variable can be available at www.cdc.gov/nchs/nhanes. According to the guidelines of American Urological Association, testosterone deficiency was defined as the total testosterone < 300 ng/dL [21].

IR was assessed with the HOMA-IR formula, and HOMA-IR was calculated as multiplied FPG (mmol/L) by FINS (IU/L) divided by 22.5 [22]. The formula for calculating METS-IR was expressed as follows: Ln [(2 × FPG (mg/dL)) + fasting TG (mg/dL)] × BMI (kg/m2))/(Ln [HDL-c(mg/dL)]) [15]. TyG index was calculated as follows: TyG = Ln [fasting TG (mg/dL) × FPG (mg/dL)/2] [23].

### Statistical analysis

Patients were divided into quartiles based on the METS-IR levels (Q1: ≤ 35.24; Q2: 35.24–41.85; Q3: 41.85–50.05; Q4: ≥ 50.05). Normal distribution was evaluated by determining skewness with a Kolmogorov–Smirnov test. The normality of continuous variables was assessed and expressed as either median and interquartile range or mean ± SD. To evaluate the disparities among quartile groups, the Kruskal–Wallis H test was employed for continuous variables, while chi-square tests were utilized for categorical variables. Through Spearman’s correlation analysis, the correlation between METS-IR and metabolic risk factors was investigated. To examine the relationship between METS-IR and testosterone, a regression model analysis was conducted, with β values and 95% confidence intervals as indicators. Meanwhile, three models were adopted: Model I without any adjustments, Model II with age and race adjustments, and Model III with age, race, SBP, DBP, total energy intake, HOMA-IR, HbA1c, E2, SHBG, ALT, AST, serum creatinine, serum uric, albumin, DM, moderate physical activity, education level adjustments.

Subgroup analysis was performed to categorize patients based on age, race, BMI, DM, moderate physical activity and education level. In addition, the smooth curve fitting and generalized additive model were adopted to identify potential nonlinear relationship between METS-IR and testosterone levels. ROC curve analysis was carried out to assess the diagnostic effectiveness of METS-IR, TG/HDL, TyG, BMI and HOMA-IR in detecting testosterone deficiency. The statistical analysis was conducted using EmpowerStats software and R, with significant difference of (P < 0.05).

## Results

### Baseline characteristics

A total of 2082 participants aged from 20 to 80 were included in this study, and the prevalence of testosterone deficiency was 18.7%. Table 1 shows the weighted population characteristics of participants by METS-IR quartiles. Compared with the bottom quartile, those in the top METS-IR quartile had a higher prevalence of testosterone deficiency, DM, as well as increased levels of BMI, waist circumference, ALT, uric acid, HbA1c, TC, TG and LDL-C. In contrast, their SHBG, HDL-C, testosterone and albumin levels were lower together with the proportion of moderate activity (P < 0.01) (Table 1).

**Table 1:** Weighted characteristics of the study population based on METS-IR quartiles

Characteristic	Q1	Q2	Q3	Q4	P value
N	520	521	521	520	
Age, years	46.6 ± 19.2	51.8 ± 17.3	50.7 ± 16.9	48.6 ± 15.7	0.076
Race, %					< 0.001
Mexican American	9.2	13.6	18.0	18.1	
Other Hispanic	8.5	9.6	14.8	11.3	
Non-Hispanic White	39.8	42.4	38.6	46.9	
Non-Hispanic Black	20.8	18.8	16.3	16.3	
Other Race	21.7	15.5	12.3	7.3	
Moderate physical activities, %					< 0.001
Yes	44.0	45.1	39.0	32.1	
No	56.0	54.9	61.0	67.9	
Diabetes					< 0.001
Yes	3.1	9.2	10.2	17.9	
No	96.9	90.8	89.8	82.1	
Education level					0.484
Less than high school	22.1	21.3	25.1	22.5	
High school or above	77.9	78.7	74.9	77.5	
Testosterone deficiency, %	5.2	13.1	18.8	37.9	< 0.001
Body mass index, Kg/m^2^	22.4 ± 2.2	26.3 ± 2.0	29.4 ± 2.2	36.1 ± 6.1	< 0.001
Waist circumference, cm	84.9 ± 8.1	96.0 ± 7.2	103.6 ± 7.7	118.8 ± 14.2	< 0.001
Systolic blood pressure, mmHg	124 ± 18	125 ± 18	126 ± 18	128 ± 16	< 0.001
Diastolic blood pressure, mmHg	68 ± 13	69 ± 13	71 ± 13	73 ± 14	< 0.001
Total energy intake, kcal/day	2429 ± 1071	2307 ± 983	2356 ± 1052	2473 ± 1146	0.518
Hemoglobin A1c, mmol/L	5.4 ± 0.5	5.6 ± 0.8	5.7 ± 1.0	6.1 ± 1.3	< 0.001
FPG, mmol/L	5.4 (5.1, 5.8)	5.7 (5.3, 6.2)	5.8 (5.4, 6.4)	6.0 (5.5, 6.9)	< 0.001
FINS, uU/mL	4.9 (3.5, 7.2)	7.7 (5.6, 10.8)	10.6 (7.5, 15.0)	16.8 (11.8, 27.8)	< 0.001
HOMA-IR	1.19 (0.83, 1.80)	2.00 (1.40, 2.87)	2.87 (1.97, 4.08)	4.86 (3.20, 8.344)	< 0.001
ALT, U/L	24.6 ± 18.0	26.1 ± 14.4	30.8 ± 20.4	34.1 ± 17.8	< 0.001
AST, U/L	27.8 ± 21.9	26.0 ± 13.8	28.2 ± 38.9	28.0 ± 11.7	0.901
Testosterone, ng/dL	580.5 ± 203.0	483.3 ± 194.2	417.4 ± 150.1	355.8 ± 164.3	< 0.001
E2, pg/ml	26.6 ± 10.5	26.2 ± 10.2	25.4 ± 8.6	27.6 ± 9.5	0.098
SHBG, ug/L	54.3 (37.6, 69.4)	41.8 (30.4, 59.4)	35.5 (25.0, 48.5)	31.0 (22.6, 44.4)	< 0.001
Albumin, g/dl	4.44 ± 0.33	4.38 ± 0.28	4.38 ± 0.28	4.29 ± 0.31	< 0.001
Creatinine, umol/L	86.3 ± 31.7	89.1 ± 26.5	88.3 ± 25.5	86.0 ± 20.2	0.840
Uric acid, umol/L	328.2 ± 68.4	351.7 ± 69.5	371.2 ± 75.7	393.3 ± 78.4	< 0.001
Total cholesterol, mmol/L	4.78 ± 0.96	4.89 ± 1.10	4.95 ± 1.14	5.01 ± 1.16	< 0.001
Triglycerides, mmol/L	0.82 (0.63, 1.08)	1.08 (0.81, 1.45)	1.43 (1.02, 2.08)	1.92 (1.28, 2.94)	< 0.001
HDL-cholesterol, mmol/L	1.62 ± 0.43	1.34 ± 0.30	1.15 ± 0.22	1.01 ± 0.25	< 0.001
LDL-cholesterol, mmol/L	2.70 ± 0.84	2.95 ± 0.94	3.04 ± 0.92	2.99 ± 0.95	< 0.001
METS-IR	30.7 ± 3.2	38.6 ± 1.9	45.7 ± 2.4	60.3 ± 10.1	< 0.001

P < 0.05 was deemed significant. *FPG* fasting plasma glucose, *FINS* fasting insulin, *HOMA* homeostatic model assessment of insulin resistance, *ALT* alanine aminotransferase, *AST* aspartate aminotransaminase, *E2* estradiol, *SHBG* sex hormone binding globulin

### Correlation between METS-IR and clinical parameters

Table 2 shows the Spearman’s correlation between METS-IR and the metabolic parameters. Notably, a positive correlation was observed between METS-IR and BMI, WC, SBP, DBP, HbA1c, TC, LDL-C, FPG, FINS, HOMA-IR, testosterone (all P < 0.001).

**Table 2:** Spearmen’s correlation of METS-IR levels with clinical and biochemical parameters

Variable	METS-IR	
	r	P
BMI	0.920	< 0.001
WC	0.843	< 0.001
SBP	0.119	< 0.001
DBP	0.174	< 0.001
HbA1c	0.265	< 0.001
TC	0.068	0.020
LDL-C	0.120	< 0.001
FPG	0.327	< 0.001
FINS	0.662	< 0.001
HOMA-IR	0.681	< 0.001
Testosterone	−0.474	< 0.001

*BMI* body mass index, *WC* waist circumference, *SBP* systolic blood pressure, *DBP* diastolic blood pressure, *HbA1c* glycosylated hemoglobin, *TC* total cholesterol, *LDL-C* Low density lipoprotein cholesterol, *FPG* fasting plasma glucose, *FINS* fasting insulin, *HOMA* homeostatic model assessment of insulin resistance

### Linear relationship between METS-IR and testosterone

**Fig. 2:**
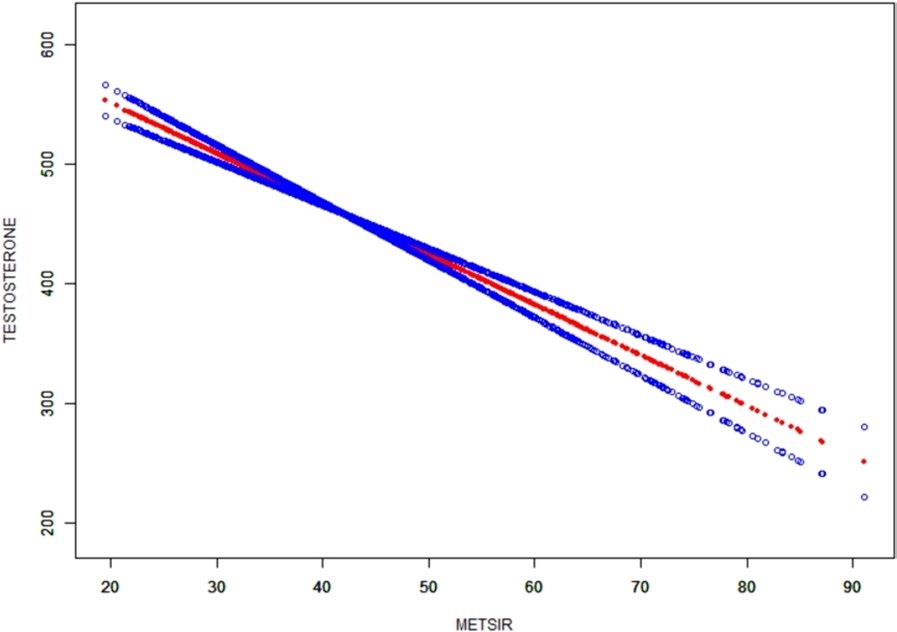
The smooth curve fit for the association between METS-IR and testosterone levels

Table 3 shows the β coefficients and corresponding 95% CI for METS-IR and testosterone in various models. In various models during adjustment, METS-IR and testosterone exhibited a significant and independent negative correlation (Model III, β = −3.88, 95% CI −4.49, −3.27; P < 0.01). In Models I and II, the β values in Q2, Q3, and Q4 groups were found to be significantly different from those in Q1 group (all P < 0.01). In Model III, after controlling for confounding variables, testosterone levels in Q2, Q3, and Q4 groups were observed to be significantly lower than that in the Q1 group. To further investigate the relationship between METS-IR and testosterone levels, a generalized additive model and smooth curve fittings were adopted (Fig. 2). Among all participants, METS-IR had a linear correlation with testosterone levels without threshold or saturation effects.

**Table 3:** Multivariate regression analysis of METS-IR with testosterone

	Model1 β (95% CI) P value	Model2 β (95% CI) P value	Model3 β (95% CI) P value
METS-IR index	−6.70 (−7.33, −6.07), < 0.001	−6.80 (−7.43, −6.17), < 0.001	−3.88 (−4.49, −3.27), < 0.001
CMI quartile			
Q1	Reference	Reference	Reference
Q2	−97.15 (−118.94, −75.37), < 0.001	−89.48 (−111.16, −67.79), < 0.001	−29.45(−45.07, −13.83), < 0.001
Q3	−163.08 (−184.86, −141.29), < 0.001	−158.16 (−179.95, −136.37), < 0.001	−53.47(−70.23, −36.72), < 0.001
Q4	−224.66 (−246.46, −202.87), < 0.001	−223.53 (−245.34, −201.72), < 0.001	−96.77 (−115.96, −77.58), < 0.001
P for trend	< 0.001	< 0.001	< 0.001

Model I: none covariates were adjusted; Model II: age and race were adjusted; Model III: age, race, SBP, DBP, total energy intake, HOMA-IR, HbA1c, E2, SHBG, ALT, AST, serum creatinine, serum uric, albumin, diabetes, moderate physical activities, education level

### Subgroup analysis

**Fig. 3:**
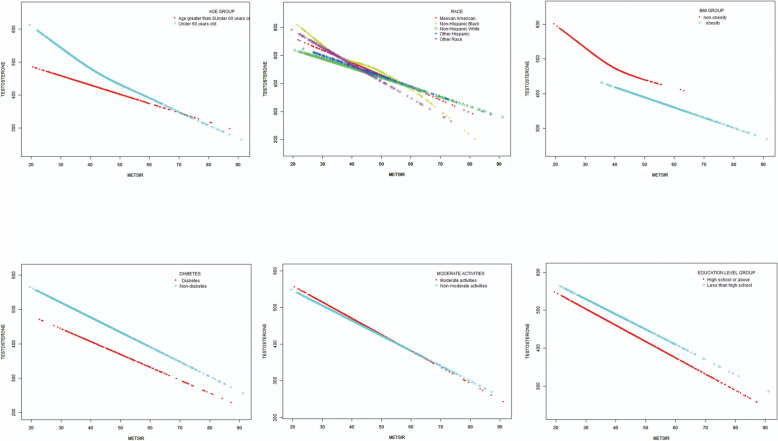
The association between METS-IR and testosterone levels stratified by age, race, BMI, diabetes, moderate physical activity and education level

The impact of subgroups on the relationship between METS-IR and testosterone levels was assessed through subgroup analyses (Table 4). As indicated by the results, all the p values in subgroups were below 0.005. METS-IR was independently correlated with testosterone levels, remaining consistent regardless of age, race, BMI, DM, moderate physical activity and education level. In the examination of the non-linear relationship through smooth curve fittings, the negative correlation between METS-IR and testosterone levels was found in all groups (Fig. 3).

**Table 4:** Association between METS-IR and testosterone stratified by age, race, BMI, diabetes, moderate activities and Education level

	β (95%CI) p value	P for interaction
Stratified by age		0.443
Age < 60 years old	−3.99 (−4.74, −3.25), < 0.001	
Age ≥ 60 years old	−3.92 (−5.16, −2.69), < 0.001	
Race		0.485
Mexican American	−3.49 (−4.75, −2.23), < 0.001	
Other Hispanic	−2.87 (−4.59, −1.15), 0.001	
Non-Hispanic White	−3.57 (−4.54, −2.61), < 0.001	
Non-Hispanic Black	−4.45 (−6.21, −2.69), < 0.001	
Other Race	−4.98 (−6.70, −3.27), < 0.001	
Stratified by BMI		0.562
BMI < 30 kg/m^2^	−3.41 (−4.63, −2.19), < 0.001	
BMI ≥ 30 kg/m^2^	−2.93 (−3.95, −1.92), < 0.001	
Stratified by diabetes		0.496
Yes	−3.12 (−4.78, −1.46), < 0.001	
No	−3.82 (−4.50, −3.13), < 0.001	
Stratified by moderate activities		0.919
Yes	−4.03 (−5.17, −2.89), < 0.001	
No	−3.80 (−4.53, −3.08), < 0.001	
Stratified by education level		0.090
Less than high school	−3.12 (−4.39, −1.85), < 0.001	
High school or above	−4.20 (−4.90, −3.49), < 0.001	

Age, race, diabetes, moderate activities, education level (not adjusted for in the subgroup analyses), SBP, DBP, total energy intake, HOMA-IR, HbA1c, E2, SHBG, ALT, AST, serum creatinine, serum uric and albumin were adjusted

### The predictive value of METS-IR for testosterone deficiency

**Fig. 4:**
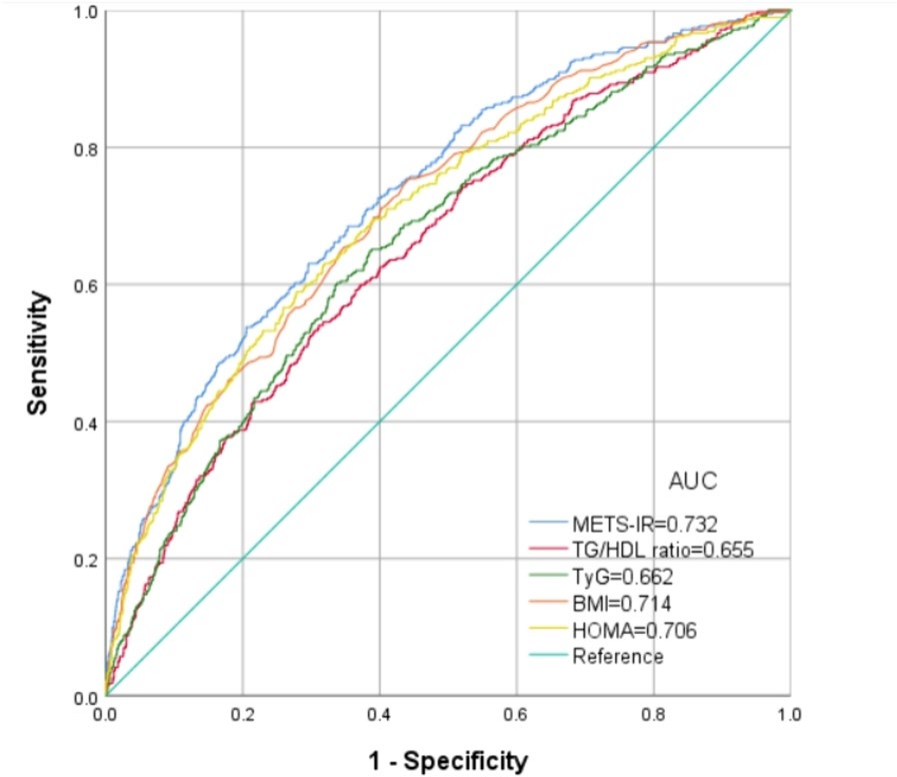
ROC analysis of METS-IR, TG/HDL ratio, TyG, BMI and HOMA-IR to identify NAFLD risk

Figure 4 shows the ROC of METS-IR, TG/HDL, TyG, BMI and HOMA-IR to diagnose testosterone deficiency. It can be observed from Table 5 that the AUC for METS-IR in the ROC analysis was 0.732 (95% CI 0.705–0.760), which was considerably higher than that of TG/HDL, BMI, TyG and HOMA-IR (P < 0.001).

**Table 5:** The results of ROC analysis of METS-IR, TG/HDL, BMI and HOMA-IR for the diagnosis of testosterone deficiency

Nutritional indices	Cut-off	Sensitivity (%)	Specificity (%)	AUC	95% CI
METS-IR	49.29	54.1	79.6	0.732	0.705–0.760
TG/HDL	0.319	54.1	68.9	0.655	0.625–0.685
TyG	8.01	60.3	66.4	0.662	0.632–0.692
BMI	27.65	75.6	55.7	0.714	0.686–0.743
HOMA-IR	3.05	63.0	68.1	0.706	0.677–0.736

## Discussion

According to our results, high METS-IR was negatively correlated with serum testosterone levels in American male adults, and exhibited a linear relationship. At a mean time, the results of subgroup analyses were stable across age, race, BMI, DM, moderate physical activity and education level. As shown in the ROC analysis, the METS-IR index exhibited superior predictive capabilities for testosterone deficiency in comparison to TG/HDL, TyG, BMI and HOMA-IR. To the best of our knowledge, this is the first study for investigating the relationship between METS-IR and testosterone levels.

The potential risk factors associated with a deficiency or decrease in testosterone levels encompass advanced obesity, age, DM and hyperlipidemia. IR is a crucial physiological mechanism in metabolic disorders, as well as a significant independent predictor of testosterone deficiency or decrease. Currently, the reciprocal relationship between IR and testosterone deficiency has been confirmed. IR is capable of diminishing testosterone levels, which, in turn, can contribute to obesity and IR [14]. Tsai et al. conducted a study involving 221 middle-aged males without DM, measuring testosterone levels. The findings from the multivariate analysis revealed a significant inverse correlation between testosterone and insulin, HOMA-IR index and C-peptide [24]. This study also observed a significant positive correlation between METS-IR and HOMA-IR, which is the key driving factor for the decrease of testosterone.

In recent years, the METS-IR index has been recommended as a viable alternative indicator for assessing insulin resistance. Based on a comprehensive cohort study, Lee et al. demonstrated the superiority of METS-IR over HOMA-IR in predicting the incidence of NAFLD. Furthermore, the findings suggest that METS-IR may serve as a more accurate index for assessing IR in comparison to HOMA-IR [16]. Through a large-scale epidemiological study, Liu et al. observed a significant correlation between elevated METS-IR levels and an increased risk of hypertension [18]. Additionally, in a community-based population without cardiovascular disease, a J-shaped relationship was identified between METS-IR and subclinical myocardial injury [25]. As indicated by the findings from Wu et al., METS-IR is a substantial prognosticator for the presence and severity of coronary heart disease (CHD), which and can potentially serve as a benchmark for the prevention and management of CHD [26]. Furthermore, the studies have revealed a strong correlation between METS-IR and various risk factors for stroke, including atherosclerosis, early renal insufficiency and hyperuricemia [27–31]. The findings of this study were aligned with previous research, indicating a notable correlation between METS-IR and multiple factors, such as BMI, WC, SBP, DBP, HbA1c, TC, LDL-C, FPG, FINS, and HOMA-IR. While underscoring the potential utility of METS-IR in clinical contexts, these findings call for further dissemination and exploration.

Nonetheless, the mechanism relationship between METS-IR and the decrease of testosterone levels in males remains uncertain, which may be attributed to the following factors: firstly, METS-IR serves as a composite index encompassing blood lipid metabolism, glucose metabolism and obesity. An atypical index frequently signifies hyperlipemia, hyperglycemia and obesity. It has been demonstrated that the insulin can regulate testosterone levels by stimulating the expression of gonadotropin-releasing hormone (GnRH) nerves in hypothalamus, thereby promoting the secretion of GnRH [32]. Conversely, hyperglycemia can diminish the expression of mitochondrial acetylase 3, triggering impaired mitochondrial function and insulin receptor damage in hypothalamic neurons, which can consequently lead to a reduction in the expression of GnRH genes and proteins within neurons, thereby inhibiting GnRH neurons and causing a decrease in testosterone levels [33]. Secondly, aromatase, primarily expressed in adipocytes, plays a crucial role in converting testosterone to estradiol in peripheral tissues. The augmentation of adipocytes in obese individuals has resulted in an elevation of aromatase expression, thereby triggering an increased conversion of testosterone in peripheral tissues [34]. Thirdly, triglyceride levels have an inverse relationship with testosterone concentrations [35]. Furthermore, obesity serves as a prevalent etiological factor for testosterone decrease. In obese males, mechanisms associated with leptin resistance and inflammatory factors contribute to suppressing hypothalamic pituitary–gonadal axis function, ultimately leading to diminished testosterone production [36, 37].

In this study, it has been determined that METS-IR exhibits superior predictive capabilities compared to TG/HDL ratio, HOMA-IR and TyG in relation to the development of testosterone deficiency in American male adults. These findings are aligned with previous investigations that compare various IR markers in different diseases. As claimed by Bello-Chavolla et al., METS-IR displayed a notably higher diagnostic performance for incident T2DM in Mexican subjects in contrast with the TyG and TG/HDL-C ratio [15]. Furthermore, an examination exploring the correlations of the METS-IR, TyG and TG/HDL-C ratio with hypertension revealed solely that the METS-IR is significantly associated with hypertension [18]. Pan et al. conducted a cohort study involving 7291 participants aged 40 or above, demonstrating that METS-IR has a greater predictive capability for major adverse cardiac events compared to other indices such as TyG-BMI, HOMA-IR, TyG-WHtR, TyG, and TyG-WC [38].

Notably, METS-IR was independently correlated with testosterone levels, which was stable across different subgroups. Given that HOMA-IR is susceptible to external influences such as exogenous insulin administration in individuals with DM [39], it is suggested that METS-IR may serve as a viable alternative in clinical settings for assessing testosterone decrease or deficiency.

The advantage of this study lies in that the subjects have been well characterized based on a large population and subgroup analyses were conducted to check whether there were differences between METS-IR and testosterone among different groups of population, thereby improving the reliability of the results. However, this study also has some limitations. Firstly, the unavailability of gonadotropin data within the NHANES database hinders our ability to identify the specific type of hypogonadism. Secondly, because of the cross-sectional design, caution should be used in causal and temporal interpretations. Therefore, the generalizability of the findings is limited to Americans. Lastly, the lack of data on testicular volume prevents its inclusion as a covariate in the analysis.

## Conclusion

To conclude, a consistent and significant inverse correlation between METS-IR and testosterone levels was observed in the study on a nationally representative sample of American male adults. Furthermore, METS-IR demonstrated superior predictive capability for testosterone deficiency in comparison to other indicators, such as TyG, TG/HDL ratio, BMI, and HOMA-IR index in this group of population. However, this relationship needs to be validated in larger cohorts through future investigation.

## Data Availability

The datasets generated and analysis during the current study are available in the NHANES, www.cdc.gov/nchs/NHANEs/

## References

[1] Tyagi V, Scordo M, Yoon RS, Liporace FA, Greene LW (2017). Revisiting the role of testosterone: are we missing something?. Rev Urol.

[2] Muller MN (2017). Testosterone and reproductive effort in male primates. Horm Behav.

[3] Shigehara K, Izumi K, Kadono Y, Mizokami A (2021). Testosterone and bone health in men: a narrative review. J Clin Med.

[4] Saad F, Rohrig G, von Haehling S, Traish A (2017). Testosterone deficiency and testosterone treatment in older men. Gerontology.

[5] Garcia-Cruz E, Alcaraz A (2020). Testosterone deficiency syndrome: diagnosis and treatment. Actas Urol Esp (Engl Ed).

[6] Rotter I, Wiatrak A, Ryl A, Kotfis K, Ciosek Z, Laszczynska M, Sipak-Szmigiel O, Szylinska A (2020). The relationship between selected bioelements and depressiveness associated with testosterone deficiency syndrome in aging men. Medicina (Kaunas).

[7] Kelly DM, Jones TH (2013). Testosterone: a metabolic hormone in health and disease. J Endocrinol.

[8] Basaria S (2014). Male hypogonadism. Lancet.

[9] Halpern JA, Brannigan RE (2019). Testosterone deficiency. JAMA.

[10] Dhindsa S, Prabhakar S, Sethi M, Bandyopadhyay A, Chaudhuri A, Dandona P (2004). Frequent occurrence of hypogonadotropic hypogonadism in type 2 diabetes. J Clin Endocrinol Metab.

[11] Corona G, Bianchini S, Sforza A, Vignozzi L, Maggi M (2015). Hypogonadism as a possible link between metabolic diseases and erectile dysfunction in aging men. Hormones (Athens).

[12] P. Souteiro, S. Belo, S.C. Oliveira, J.S. Neves, D. Magalhaes, J. Pedro, R. Bettencourt-Silva, M.M. Costa, A. Varela, J. Queiros, P. Freitas, D. Carvalho, A. Group (2018). Insulin resistance and sex hormone-binding globulin are independently correlated with low free testosterone levels in obese males. Andrologia.

[13] Kupelian V, Page ST, Araujo AB, Travison TG, Bremner WJ, McKinlay JB (2006). Low sex hormone-binding globulin, total testosterone, and symptomatic androgen deficiency are associated with development of the metabolic syndrome in nonobese men. J Clin Endocrinol Metab.

[14] Pivonello R, Menafra D, Riccio E, Garifalos F, Mazzella M, de Angelis C, Colao A (2019). Metabolic disorders and male hypogonadotropic hypogonadism. Front Endocrinol (Lausanne).

[15] Bello-Chavolla OY, Almeda-Valdes P, Gomez-Velasco D, Viveros-Ruiz T, Cruz-Bautista I, Romo-Romo A, Sanchez-Lazaro D, Meza-Oviedo D, Vargas-Vazquez A, Campos OA, Sevilla-Gonzalez MDR, Martagon AJ, Hernandez LM, Mehta R, Caballeros-Barragan CR, Aguilar-Salinas CA (2018). METS-IR, a novel score to evaluate insulin sensitivity, is predictive of visceral adiposity and incident type 2 diabetes. Eur J Endocrinol.

[16] Lee JH, Park K, Lee HS, Park HK, Han JH, Ahn SB (2022). The usefulness of metabolic score for insulin resistance for the prediction of incident non-alcoholic fatty liver disease in Korean adults. Clin Mol Hepatol.

[17] Han Y, Zhou Z, Zhang Y, Zhao G, Xu B (2023). The association of surrogates of insulin resistance with hyperuricemia among middle-aged and older individuals: a population-based nationwide cohort study. Nutrients.

[18] Liu XZ, Fan J, Pan SJ (2019). METS-IR, a novel simple insulin resistance indexes, is associated with hypertension in normal-weight Chinese adults. J Clin Hypertens (Greenwich).

[19] Rattanatham R, Tangpong J, Chatatikun M, Sun D, Kawakami F, Imai M, Klangbud WK (2023). Assessment of eight insulin resistance surrogate indexes for predicting metabolic syndrome and hypertension in Thai law enforcement officers. PeerJ.

[20] Zipf G, Chiappa M, Porter KS, Ostchega Y, Lewis BG, Dostal J (2013). National health and nutrition examination survey: plan and operations, 1999–2010. Vital Health Stat.

[21] Mulhall JP, Trost LW, Brannigan RE, Kurtz EG, Redmon JB, Chiles KA, Lightner DJ, Miner MM, Murad MH, Nelson CJ, Platz EA, Ramanathan LV, Lewis RW (2018). Evaluation and management of testosterone deficiency: AUA guideline. J Urol.

[22] Wallace TM, Levy JC, Matthews DR (2004). Use and abuse of HOMA modeling. Diabetes Care.

[23] Liu XC, He GD, Lo K, Huang YQ, Feng YQ (2020). The triglyceride-glucose index, an insulin resistance marker, was non-linear associated with all-cause and cardiovascular mortality in the general population. Front Cardiovasc Med.

[24] Tsai EC, Matsumoto AM, Fujimoto WY, Boyko EJ (2004). Association of bioavailable, free, and total testosterone with insulin resistance: influence of sex hormone-binding globulin and body fat. Diabetes Care.

[25] Wang Z, Li W, Li J, Liu N (2022). The nonlinear correlation between a novel metabolic score for insulin resistance and subclinical myocardial injury in the general population. Front Endocrinol (Lausanne).

[26] Wu Z, Cui H, Li W, Zhang Y, Liu L, Liu Z, Zhang W, Zheng T, Yang J (2022). Comparison of three non-insulin-based insulin resistance indexes in predicting the presence and severity of coronary artery disease. Front Cardiovasc Med.

[27] Cai XT, Zhu Q, Liu SS, Wang MR, Wu T, Hong J, Hu JL, Li N (2021). Associations between the metabolic score for insulin resistance index and the risk of type 2 diabetes mellitus among non-obese adults: insights from a population-based cohort study. Int J Gen Med.

[28] Ding L, Gao YH, Li YR, Huang YF, Wang XY, Qi X (2020). Metabolic score for insulin resistance is correlated to adipokine disorder and inflammatory activity in female knee osteoarthritis patients in a chinese population. Diabetes Metab Syndr Obes.

[29] Bello-Chavolla OY, Antonio-Villa NE, Vargas-Vazquez A, Martagon AJ, Mehta R, Arellano-Campos O, Gomez-Velasco DV, Almeda-Valdes P, Cruz-Bautista I, Melgarejo-Hernandez MA, Munoz-Hernandez L, Guillen LE, Garduno-Garcia JJ, Alvirde U, Ono-Yoshikawa Y, Choza-Romero R, Sauque-Reyna L, Garay-Sevilla ME, Malacara-Hernandez JM, Tusie-Luna MT, Gutierrez-Robledo LM, Gomez-Perez FJ, Rojas R, Aguilar-Salinas CA (2019). Prediction of incident hypertension and arterial stiffness using the non-insulin-based metabolic score for insulin resistance (METS-IR) index. J Clin Hypertens (Greenwich).

[30] Zhang M, Liu D, Qin P, Liu Y, Sun X, Li H, Wu X, Zhang Y, Han M, Qie R, Huang S, Li Y, Wu Y, Yang X, Feng Y, Zhao Y, Hu D, Hu F (2021). Association of metabolic score for insulin resistance and its 6-year change with incident type 2 diabetes mellitus. J Diabetes.

[31] Lee JH, Kwon YJ, Park K, Lee HS, Park HK, Han JH, Ahn SB (2022). Metabolic score for insulin resistance is inversely related to incident advanced liver fibrosis in patients with non-alcoholic fatty liver disease. Nutrients.

[32] Christian CA, Moenter SM (2010). The neurobiology of preovulatory and estradiol-induced gonadotropin-releasing hormone surges. Endocr Rev.

[33] Morelli A, Comeglio P, Sarchielli E, Cellai I, Vignozzi L, Vannelli GB, Maggi M (2013). Negative effects of high glucose exposure in human gonadotropin-releasing hormone neurons. Int J Endocrinol.

[34] Rubinow KB (2017). Estrogens and body weight regulation in men. Adv Exp Med Biol.

[35] Sung SH, Kim NH, Hong SP, Lee JK, Choi SJ (2019). Associations of metabolic syndrome with total testosterone and homocysteine levels in male korean workers. Endocrinol Metab (Seoul).

[36] Sun K, Wang C, Lao G, Lin D, Huang C, Li N, Li L, Li F, Xiao H, Yan L (2020). Lipid accumulation product and late-onset hypogonadism in middle-aged and elderly men: results from a cross-sectional study in China. BMJ Open.

[37] Ghanim H, Aljada A, Daoud N, Deopurkar R, Chaudhuri A, Dandona P (2007). Role of inflammatory mediators in the suppression of insulin receptor phosphorylation in circulating mononuclear cells of obese subjects. Diabetologia.

[38] Pan L, Zou H, Meng X, Li D, Li W, Chen X, Yang Y, Yu X (2023). Predictive values of metabolic score for insulin resistance on risk of major adverse cardiovascular events and comparison with other insulin resistance indices among Chinese with and without diabetes mellitus: results from the 4C cohort study. J Diabetes Investig.

[39] Wang L, Cong HL, Zhang JX, Hu YC, Wei A, Zhang YY, Yang H, Ren LB, Qi W, Li WY, Zhang R, Xu JH (2020). Triglyceride-glucose index predicts adverse cardiovascular events in patients with diabetes and acute coronary syndrome. Cardiovasc Diabetol.

